# Hairy Cell Leukemia: Where Are We in 2023?

**DOI:** 10.1007/s11912-023-01419-z

**Published:** 2023-04-25

**Authors:** Andres Mendez-Hernandez, Krishna Moturi, Valeria Hanson, Leslie A. Andritsos

**Affiliations:** 1 Division of Hematology and Oncology, University of New Mexico Comprehensive Cancer Center, 1 University of New Mexico, 1201 Camino de Salud, NE Albuquerque, NM 87102 USA; 2 Division of Internal Medicine, University of New Mexico School of Medicine, MSC08 4720 1 UNM, Albuquerque, NM 87131-0001 USA

**Keywords:** Hairy cell leukemia, Rituximab for hairy cell leukemia, Bone marrow transplant for hairy cell leukemia, BRAF inhibitors for hairy cell leukemia, Cladribine, Pentostatin, Moxetumomab, Bendamustine, Ibrutinib, Dabrafenib

## Abstract

**Purpose of Review:**

This article summarizes the current state of knowledge of hairy cell leukemia (HCL) regarding presentation, diagnosis, therapy, and monitoring, including perspectives on emergent therapies.

**Recent Findings:**

Over the past decade, there has been enormous progress in the understanding of the biology of HCL which has led to the development of novel therapeutic strategies. The maturation of data regarding existing management strategies has also lent considerable insight into therapeutic outcomes and prognosis of patients treated with chemo- or chemoimmunotherapy. Purine nucleoside analogs remain the cornerstone of treatment, and the addition of rituximab has deepened and prolonged responses in the upfront and relapsed setting. Targeted therapies now have a more defined role in the management of HCL, with BRAF inhibitors now having a potential in the first-line setting in selected cases as well as in relapse. Next-generation sequencing for the identification of targetable mutations, evaluation of measurable residual disease, and risk stratification continue to be areas of active investigation.

**Summary:**

Recent advances in HCL have led to more effective therapeutics in the upfront and relapsed setting. Future efforts will focus on identifying patients with high-risk disease who require intensified regimens. Multicenter collaborations are the key to improving overall survival and quality of life in this rare disease.

## Introduction

Hairy cell leukemia (HCL) is a rare hematological malignancy that arises from late-activated post-germinal center memory B-cells [1••]. The hallmark of HCL is the presence of medium-sized mature lymphocytes with “hairy” projections in the bone marrow, spleen, and sometimes extramedullary tissues. Peripheral blood involvement is less common. [2]. HCL represents 2% of new leukemias and is more frequent in men than women, with a median age at diagnosis of approximately 52–63 in men and 51–59 in women [1••, 3]. Common clinical findings at the time of diagnosis include pancytopenia, splenomegaly, and increased risk of infection; monocytopenia is nearly universal, and the mechanism of this remains elusive. Extramedullary involvement such as lymphadenopathy and bone involvement are uncommon at diagnosis but may be seen more frequently in the relapsed setting [4].

The HCL landscape is rapidly changing, and advances in this disease warrant a review of recent data relevant to the clinical hematologist. In this article, we will provide a concise review of advances over the last 10 years, focusing on the diagnosis, first-line treatment, outcomes in relapsed/refractory disease, and management in patients with active infections or with comorbidities. This article will focus on classical HCL, given that previously termed HCL-variant was reclassified by the World Health Organization in 2022 as “Splenic B-cell Lymphoma/Leukemia with Prominent Nucleoli” (SBLPN, [5]).

## Diagnostic Criteria for HCL: Review of Current HCLF Guidelines

In 2017, Grever et al. published the first consensus guidelines for the diagnosis and management of HCL [2]. These guidelines provide a comprehensive review of requirements for HCL diagnosis, therapeutic options, and response assessments. Since the time of the initial publication, additional cytogenetic and molecular discoveries have emerged. Therefore, the following will provide a review of current recommendations for diagnostic testing as well as potential additional testing to be performed in select circumstances.

### Diagnosis

According to the consensus guidelines, a diagnosis of HCL is suggested by the clinical presentation of the patient and confirmed by laboratory findings including a complete blood count with peripheral blood smear review, a bone marrow aspiration, and trephine biopsy with the assessment of bone marrow morphology, immunohistochemistry, flow cytometry, and testing for the identification of the BRAF V600E mutation. In cases where the diagnosis remains obscure or does not meet the consensus criteria, stains for annexin-1 or PD-1 could be helpful [2, 6]. As stated in the guidelines, imaging as part of the initial work-up is optional and recommended in select cases, such as chest X-rays in patients with suspected pneumonia or CT or ultrasound to evaluate for organomegaly. PET/CT may have a role in the evaluation, especially if extramedullary disease is suspected; however, this is an area of ongoing investigation as there is no currently accepted standard uptake variable (SUV) in HCL [7–9].

Rarely, HCL presents without bone marrow involvement or splenomegaly. Extramedullary HCL can mimic lymphoma and/or myeloma, commonly in the form of soft tissue masses or skeletal lesions. These lesions are usually lytic although osteoblastic lesions can also be seen. Treatment would not differ from standard HCL except in that bone stability would need to be considered in cases of skeletal HCL and assessment of response to therapy would require repeat imaging, possibly with F-FDG PET/CT imaging if this were the only positive finding prior to treatment [7, 8, 10–12].

## Immunophenotyping, Genetics, and Molecular Updates

### Immunophenotype

HCL is characterized by bone marrow or extramedullary involvement by an abnormal population of light chain restricted B-cells with a distinct immunophenotype comprised of CD11c, CD103, CD123, and CD25 according to the diagnostic score proposed by Matutes et al. [1••, 13]. HCL cells additionally co-express CD19, CD20, CD22, and CD200 and are classically negative for CD5, CD10, CD23, and CD27. However, similar to other hematological malignancies, aberrant expression of atypical markers or loss of expression of typical markers may be seen. Cases of aberrant expression of CD5, CD10, and absence of CD123 have been reported [14–16]. In these cases where classical HCL is strongly suspected, additional immunochemical stains for expression of annexin-1, VE1 (BRAFV600E), or PCR for the BRAF-V600E mutation can help clarify the diagnosis [4]. The immunophenotypic profile of HCL may also provide insight into prognosis. The presence of CD38, expressed in 7% of cases of HCL in a case series [4] but estimated to be as high as 1/3 of patients, was associated with substantially shorter time to next treatment (TTNT) with a difference of 3 or more years compared to those who were CD38 negative [17]. Finally, CD25 has traditionally been considered a diagnostic feature of HCL, and the absence of CD25 is sometimes viewed as excluding a diagnosis of HCL. However, it should be noted CD25 is lost following treatment due to therapy-related alterations and should not be relied upon for the diagnosis of HCL in patients who have already initiated therapy [18].

### Molecular and Genetic Features

In 2011, Tiacci et al. reported the crucial observation that the BRAF V600E mutation was present in 100% of HCL patients and negative in all patients with other lymphoproliferative disorders [19]. The BRAF V600E mutation, which is also seen in solid tumors such as melanoma and lung cancer, has since been shown to be present in the vast majority of cases of HCL and constitutes an activating mutation which translates into increased proliferation and survival of malignant cells. Patients with a classical immunophenotype but who lack BRAF V600E may harbor alternative BRAF mutations [20, 21•]. Additional driver mutations in the MAP2K1 pathway may be present, and one case series demonstrated MAP2K1 mutations in one-third of those who were unmutated for BRAF [1••, 21•, 22]. Following the discovery of BRAF V600E mutations in HCL, other investigators identified alterations in smaller numbers of patients, including KLF2 (23%) and CDKN1B (7.5%). Finally, given its significance in other lymphoid malignancies, mutations of TP53 have been identified in HCL but occur at a lower frequency, ranging from 0 to 27% [22]. This finding has been associated with an unmutated IGHV status, resistance to cladribine, and shorter event-free survival [23]. Patients who are unresponsive to PA may benefit from TP53 status assessment [1••].

## Risk Stratification and Prognosis

Unlike the majority of hematological malignancies, there are no standardized risk-stratification guidelines in HCL, and this remains an area of needed investigation. HCL has been associated with worse outcomes in patients with splenomegaly, higher beta-2 microglobulin, leukocytosis (> 10 × 10^9^/L), and an elevated hairy cell count (> 5 × 10^9^/L). In contrast, in a retrospective study done by Maral et al., the only prognostic parameter was an elevated LDH level at the time of diagnosis, with an optimal cutoff of 200.5 IU for a sensitivity of 73.3% and specificity of 61.2%, predicting a higher risk of recurrence and shorter PFS; no other parameters correlated with adverse outcomes [24].

Maître et al. analyzed the most common genetic alterations in HCL and found that patients with MAP2K1 mutations had a shorter TTNT and progression-free survival in comparison to other mutations [21•]. Similarly, around 12% of HCL patients will have unmutated IGHV which correlates with resistance to cladribine, rapid clinical progression, and shorter OS [1••, 25].

Finally, male versus female sex has also been found to have prognostic value. A recent study utilizing data from the HCL Patient Data Registry [3] found no significant differences in response rates between male and female patients but found significantly longer median time to next treatment in females compared to males (17.6 years vs. 8 years), a finding which persisted after adjusting for response rates and BRAF status. The reasons for these differences remain unclear, and additional studies are underway [3].

## Response Assessment and Measurable Residual Disease

Post-treatment response should include a complete physical exam with attention to organomegalies along with a peripheral blood examination and a bone marrow evaluation. The bone marrow biopsy should be delayed until 4–6 months post-cladribine therapy and approximately 2 weeks after completion of the final dose of pentostatin [26]. A complete remission is defined as near normalization of peripheral blood counts: hemoglobin > 11 g/dL, platelets > 100,000 µL, and ANC > 1500/µL along with regression of splenomegaly by physical exam and morphological absence of hairy cells in bone marrow and peripheral blood [2]. In patients who achieve a CR, some trials have used measurable residual disease (MRD) as an endpoint [27••].

MRD, although it can guide treatment in other diseases, remains controversial in HCL. HCL patients who have undetectable MRD (uMRD) have a longer median relapse-free and treatment-free survival; however, MRD positivity would not impact subsequent management as there is also evidence of long-term relapse-free survival in some that still harbor HCL cells at the end of therapy [28, 29]. Currently, MRD is being used more as a measure of the depth of response and as a predictor of duration of remission, especially in clinical trials, rather than as a parameter to be monitored after therapy. There is likewise no consensus on the method of measurement, with a recent article from an expert panel describing MRD testing via methods including multi-parameter flow cytometry (MFC), allele-specific PCR for mutant BRAF, and IHC in marrow aspirate samples, with MFC and PCR being significantly more sensitive. The panel suggested all trials in the relapse setting should include MRD as part of response assessment [30•]. However, these recommendations have not yet translated to routine clinical practice given the lack of consensus regarding the method of testing and parameters for positivity.

A partial response (PR) is defined as near normalization of the peripheral blood counts with at least 50% of improvement in organomegaly and marrow findings. These patients can be observed provided they remain asymptomatic with appropriate blood counts above treatment threshold. Management of those who have a partial response and remain symptomatic is debatable as some authors have used a second course of PA, an alternate PA, and/or rituximab [2]. However, consideration for the referral of these patients to a HCL Center of Excellence for additional evaluation may be considered if a second course of PA is under consideration given the rarity of this scenario.

## Indications for Treatment

The majority of patients will require treatment at the time of diagnosis as a result of peripheral blood cytopenias or splenomegaly. According to consensus guidelines, initiation of treatment is guided by the presence of at least one of the following: hemoglobin < 11 g/dL, platelets < 100,000/µL, or absolute neutrophil count (ANC) < 1000/µL. Based on these thresholds, a small percentage of patients (approximately 10%) will not need to be treated at the time of diagnosis, and active surveillance is appropriate until intervention is needed [1••, 2].

## Initial Therapy

While HCL cannot be cured with currently available therapies, highly effective treatments are available for eligible patients. The backbone of therapy remains a purine nucleoside analog (PA), either in the form of cladribine or pentostatin which have traditionally been used as monotherapies. Although these drugs have not been directly compared in randomized trials, they appear to be equally effective for the achievement of CR and PR (76–83% and 31–33%, respectively) [1••, 2]. However, recent studies have changed the paradigm of first-line treatment for HCL given the effectiveness of the addition of rituximab to a PA for initial therapy. Rituximab has been added to first-line PA therapy either concurrently or sequentially [27••, 31]. This chemoimmunotherapy combination has been shown to achieve a CR in virtually all patients with a deeper (97% vs 24% at 6 months) and longer (94% vs 12% at 96 months) MRD-free CR when compared to delayed rituximab (i.e., starting 6 months later in MRD-positive patients) with comparable toxicity [27••, 31]. This suggests that the addition of rituximab early in the induction treatment in combination with cladribine can deepen and prolong responses, as opposed to delayed rituximab administration or cladribine monotherapy, and is a safe first-line option. Because of the outstanding and prolonged responses, PA + rituximab should be considered the standard of care in fit patients requiring first-line treatment for HCL. However, as discussed below, some patients will not be eligible for chemo-immunotherapy and will require alternative induction strategies.

## Contraindications to PA Therapy

Patients with active infections, significant renal dysfunction, pregnancy, or underlying neurological abnormalities may not be eligible initially to receive a PA. Traditionally, for this group of patients, interferon alpha has been successfully utilized to improve peripheral blood counts and can serve as a bridge to more definitive therapy once the contraindication has resolved (ORR 82% [32]). Vemurafenib, an oral BRAF inhibitor (BRAFi), has significant activity when used as either monotherapy or in combination with rituximab and is generally reserved for patients who are either refractory to chemo-immunotherapy or have relapsed. BRAFi may have a role as a first-line treatment in cases where a PA is contraindicated, such as those with serious infections and cytopenias or high risk for PA therapy complications. In a case series that included 3 treatment-naïve HCL patients with contraindications to PA, the combination of rituximab with a short duration of vemurafenib was well tolerated and achieved durable remissions. This combination is a reasonable initial therapy that can also serve as a bridge to PA in those who cannot receive a PA initially as first-line treatment and have the BRAF V600E mutation [33].

Treatment options are limited in pregnancy due to the classification of PA as class D drugs based on teratogenicity seen in animal studies. IFN has been used successfully during pregnancy, often producing partial remissions. However, it is becoming increasingly difficult to obtain due to decreased production. Splenectomy also remains an option, although it should be used as a bridging therapy until a post-partum PA can be used as it only improves blood counts but has no pathological remissions; splenectomy could also carry an increased risk of bleeding and surgical complications in late pregnancy. Data on rituximab during pregnancy remains inconclusive, with studies showing no clear pattern of anomalies related to its use [1••, 34, 35].

## Management of Relapsed HCL

Therapy for relapsed/refractory disease is contingent on treatment previously received, response to prior therapy, and timing of recurrence. In patients with an initial response of 24 months or longer, re-treatment with a PA generally with rituximab is appropriate [1••]. PA + R was used by Chihara et al. in a subset of patients with HCL in first relapse, achieving a CR, 5-y FFS, and OS of 100% and uMRD in more than half of the relapsed cohort [31]. In those patients with a response shorter than 24 months, an alternative therapy to the initial PA should be considered [2].

### BRAF/MEK Inhibitors

For patients with the BRAFV600E mutation who relapse after a PA, vemurafenib monotherapy or in combination with anti-CD20 has shown favorable results. As monotherapy, vemurafenib used in fixed, short periods has been shown to achieve a high overall response rate (ORR) (96–100%) and a CR in approximately a third of those treated; however, those with a CR remained MRD positive [36]. The addition of rituximab to vemurafenib increased the CR rate (≥87%) with the majority of those achieving uMRD, including those with a previous refractoriness to rituximab or suboptimal response to vemurafenib monotherapy. This combination led to a faster response with a shorter duration of treatment and significantly longer relapse-free survival and remains an excellent therapeutic alternative after first relapse [37•].

A retreatment course of vemurafenib +R can be given to those patients who progress after an initial fixed treatment with this combination [38], although it is expected that successive responses will be shorter in duration. The mechanism driving shorter responses remains unclear, although resistance through activating mutations of KRAS and MAP2K1 has been described in HCL [39]. MAP2K1 encodes MEK1, and the addition of a MEK inhibitor (MEKi) to a BRAFi has been found to be beneficial in relapsed HCL [40]. In this sense, some authors advocate for a short, fixed duration of treatment instead of a continuous regimen to avoid the constant selective pressure of the leukemic clone [38].

### BTK Inhibitors

Survival and proliferation of HCL cells are promoted through the B-cell receptor (BCR) pathway via downstream activation of Bruton’s tyrosine kinase (BTK). This pathway can be inhibited by the BTK inhibitor (BTKi) ibrutinib, decreasing HCL cells’ survival and growth [41]. A recent phase 2 trial recently evaluated the efficacy and safety of the BTKi ibrutinib in relapsed HCL as well as untreated SBPLN. With 37 patients enrolled and 76% having classic HCL, the ORR at 48 weeks was 36%; of those achieving a CR (7 out of 37 patients), 3 had uMRD. Longer treatment duration resulted in deeper responses. More data is needed to elucidate the role of a BTKi in relapsed HCL [42]; however, significant clinical benefit was observed with many patients remaining on therapy for a prolonged period of time.

### Bendamustine and Anti-CD20 Monoclonal Antibodies

The alkylating agent bendamustine has been used in a variety of hematological malignancies and has been demonstrated to be synergistic when combined with an anti-CD20 such as rituximab. In a small trial involving 12 patients with multiply relapsed HCL (including one SBPLN) assigned to two different doses of bendamustine in combination with rituximab for 6 cycles, the ORR was 100% with 7 patients achieving a CR and a high percentage of uMRD (100% in those assigned to the highest bendamustine dose of 90 mg/m^2^/dose) [43]. This combination should be generally reserved for patients with an appropriate bone marrow reserve [38].

Although HCL cells are brightly CD20 positive, in one study, single-agent rituximab was only able to achieve a CR in 13% of patients with relapsed disease [43]. However, this response rate may belie the clinical usefulness of the drug for disease stabilization and rituximab may be indicated as a single agent in some instances. In addition, there have been two case reports of the use of obinutuzumab in patients who were refractory or intolerant to rituximab and the drug was utilized in combination with bendamustine with encouraging results in one case, achieving uMRD [44, 45]. Larger studies of anti-CD20 both as a single agent and in combination in HCL are warranted.

### Immunotoxin Drug Conjugates

Moxetumomab pasudotox (MoP), a recombinant immunoglobulin linked to a *Pseudomonas* exotoxin targeting CD22, was initially developed as a treatment for relapsed or refractory HCL. Kreitman et al. studied MoP in patients with at least two prior systemic therapies, observing an ORR of 75% with CR being 41% and a majority achieving uMRD (approximately 82% of those achieving CR). Those that remained MRD positive had a median duration of CR of 12 months, compared to those that remained uMRD where the median duration of CR was 62.8 months. MoP was also associated with hemolytic uremic syndrome in 7.5% that responded to discontinuation of treatment, as well as cytokine release syndrome which was reversible. Unfortunately, despite its efficacy, the drug has been withdrawn from the US markets and will no longer be developed for use in HCL [46••, 47].

### Bone Marrow Transplant, Cellular Therapy, and Emergent Therapies

Recently, Chihara et al. published a case series of HCL and SBLPN patients undergoing first allogeneic hematopoietic cell transplant (HCT) in refractory/relapsed HCL. A total of 24 patients were transplanted (2 with SPLBN), and a CR was achieved in 59% of patients, with non-relapse mortality occurring in 14%. With an estimated 5-year OS of 46%, allogeneic HCT presents a potential therapeutic option for heavily pretreated patients with HCL and offers the possibility of achieving long-term OS in a significant percentage of patients [48•].

Unexplored treatment approaches are currently being tested, such as CAR T-cell therapy in relapsed HCL using autologous anti-CD22 CAR T-cells and against B-cell activating factor (BAFF), a ligand expressed by hairy cells [49, 50]. Monoclonal antibodies targeting ROR1 and ROR2, which are oncoembryonic antigens found on cancer cells and promote movement, survival, and proliferation, are also being studied [51]. The results of these trials will be eagerly awaited. In addition, a trial of encorafenib plus binimetinib is currently enrolling, as is a trial of anti-CD22 CAR T-cell immunotherapy [49, 52]. There remain unexplored potential treatments in HCL including anti-CD38 therapy such as daratumumab or isatuximab, or PD-1 inhibition given the recent discovery of high levels of PD-1 expression in HCL [6, 53].

## Long-Term Follow-Up

Following completion of therapy and assessment of clinical response with bone marrow evaluation, HCL patients should be followed periodically with routine physical examinations and peripheral blood counts. The recurrence of persistent cytopenias should prompt repeat bone marrow evaluation. However, the majority of patients will continue to do well for a prolonged period of time. Similar to CLL, some patients with HCL may suffer from autoimmune phenomenon [54], and new symptoms may require the assistance of other medical subspecialties. In addition, patients who receive PA will remain at risk of infection for up to a year following the completion of therapy and require careful monitoring. Importantly, patients with HCL appear to be at increased risk of second primary malignancies [55]. Studies have found rates of second primary malignancies ranging from 7.3 to 24%. The median time from HCL diagnosis to secondary cancer differs by age, with patients who were ≤ 40 years old at diagnosis having a median time of 19 years compared to 4 years in those > 40 years. It remains unclear if the higher risk of secondary neoplasms is from exposure to chemotherapeutic agents or from immune alterations related to the HCL pathophysiology. Skin cancers are the most frequent secondary malignancies found, although as a group visceral neoplasms and hematological malignancies are also common [55–57]. Routine cancer screening along with annual dermatology evaluation is suggested.

## Treatment Considerations During COVID-19

In consideration of the current ongoing COVID-19 pandemic, treatment options have to be tailored to the patient’s needs and have to take into consideration the patient’s environment and probability of COVID-19 exposure. Patients with hematological cancers have a similar case rate of COVID-19 but more severe disease and higher fatality rates [58]. Furthermore, this can be aggravated by treatments such as anti-CD20 antibodies which decrease the effectiveness of vaccinations for at least 6 months [59] and purine analogs that contribute to a prolonged period of immunosuppression.

Treatment with the BRAFi vemurafenib allowed successful COVID-19 vaccination within 2 months of initiation with afterwards detectable IgG antibody levels against the COVID-19 spike protein and later permitting treatment with rituximab. Vemurafenib can therefore be an option in cases where patients do not have appropriate COVID-19 antibody titers and serve as a bridge for therapies that would otherwise hinder vaccine response [60].

## Conclusion

Advances in the understanding of HCL have led to new approaches in management and improved outcomes; nonetheless, there is room for improvement. Risk stratification and MRD continue to be areas of active investigation, and additional studies of molecular and genetic predictors of outcomes will further refine future treatment recommendations. HCL remains a success story highlighting the positive impact of large multicenter clinical trials in rare diseases which in this case changed a universally fatal illness into one with a survival comparable to that of unaffected counterparts.
